# Evidence for Aerosol Transfer of SARS-CoV-2–Specific Humoral Immunity

**DOI:** 10.4049/immunohorizons.2300027

**Published:** 2023-05-09

**Authors:** Ross M. Kedl, Elena W. Y. Hsieh, Thomas E. Morrison, Gabriela Samayoa-Reyes, Siobhan Flaherty, Conner L. Jackson, Rosemary Rochford

**Affiliations:** * Department of Immunology and Microbiology, School of Medicine, University of Colorado Anschutz Medical Campus, Aurora, CO; † Department of Pediatrics, School of Medicine, University of Colorado Anschutz Medical Campus, Aurora, CO; ‡ Department of Biostatistics and Informatics, Colorado School of Public Health, University of Colorado Anschutz Medical Campus, Aurora, CO

## Abstract

Infectious particles can be shared through aerosols and droplets formed as the result of normal respiration. Whether Abs within the nasal/oral fluids can similarly be shared between hosts has not been investigated. The circumstances of the SARS-CoV-2 pandemic facilitated a unique opportunity to fully examine this provocative idea. The data we show from human nasal swabs provides evidence for the aerosol transfer of Abs between immune and nonimmune hosts.

## Introduction

The vaccines against SARS-CoV-2 have maintained remarkable efficacy against severe disease and death in those vaccinated regardless of variant emergence, Omicron included (1). Less appreciated than the systemic immunity generated by the vaccines are the high levels of Ab (IgG and IgA) found within the nasal cavity and saliva of vaccinees. This outcome is found in both humans and primates, and in response to both mRNA and protein-based vaccines (Ref. 2 and G.R. Nahass, R.E. Salomon-Shulman, G. Blacker, K. Haider, R. Brotherton, K. Teague, Y.Y. Yiu, R.E. Brewer, S.D. Galloway, P. Hansen, manuscript posted on bioRxiv, DOI: 10.1101/2021.08.22.21262168). Respiratory transmission of viral infection is proof that oral/nasal cavity constituents can be communicated through aerosols and/or respiratory droplets. As such, it would stand to reason that Ab present within the oral/nasal environment may also be aerosolized to some degree.

## Materials and Methods

### Multiplexed microsphere immunoassay

A multiplexed microsphere immunoassay (MMI) was developed using BioLegend carboxylated LEGENDplex microbeads to simultaneously quantify IgG and IgA against the spike receptor-binding domain (RBD) and nucleocapsid of the Wuhan strain of SARS-CoV-2 (Sino Biological, RBD, catalog no. 40150-D002; nucleocapsid, catalog no. 40143-MM08) and tetanus toxoid (TT) (MilliporeSigma, catalog no. 582231) as a positive control. BSA-conjugated beads were used as a negative control. Validation of RBD and TT protein-bead conjugation was performed by staining with an anti-RBD mAb (human chimeric, D002, Sino Biological, Wayne, PA) or anti-TT mAb (mouse Ab, Jackson ImmunoResearch, West Grove, PA), respectively. Beads were mixed in equal ratios (∼2000 each bead/sample well) and incubated with serum, saliva, mask eluates, or nasal swab samples into storage/running buffer (PBS containing 0.01% Tween 20, 0.05% NaN_3_, and 0.1% BSA) and rocked on a shaker plate for 60 min at room temperature and then washed. Bound IgG was detected by secondary anti-human IgG-biotin (1:3000 dilution) (SouthernBiotech, Birmingham, AL), followed by addition of streptavidin-PE (1:1000 dilution) (BD Biosciences, San Jose, CA) and anti-human F(ab′)_2_ IgA-FITC (SouthernBiotech, Birmingham, AL). The geometric mean fluorescence intensity (gMFI) of the IgG/IgA for each sample and dilution was captured with a CytoFLEX S flow cytometer (Beckman Coulter) and analyzed with FlowJo (version 10.7.1; BD Biosciences) (3, 4). Prism (version 8.4.3, GraphPad) was used to plot data.

### Human sample acquisition and analysis

Human serum, saliva, and nasal swabs were obtained (Institutional Review Board approval no. 20-1279). Surgical masks were anonymously donated by laboratory workers at the end of one work day. Punches (four) were taken from the middle of each mask and Ab was eluted as previously described (4). Briefly, a 6-mm handheld punch was used to make punches from the mask, collected in a 24-well cell culture plate with 500 μl of an elution buffer (PBS containing 0.05% Tween 20 and 0.08% NaN_3_), then placed on a platform shaker for 2 h at room temperature. The elution was collected and stored at −80°C until use in the MMI. Nasal swabs were obtained by convenience sampling both parents and their children at the Colorado Tri-County vaccine center in Aurora, CO, who were attending vaccine appointments, not limited to SARS-CoV-2. Ab from swab tips was eluted as described for dried blood spots (4). The log-transformed IgA and IgG values from the children’s samples were modeled using linear regression with a single binary covariate corresponding to high or low Ab levels from their parent. Because the distribution of the data for adult IgG gMFI was unimodal and heavily right skewed, a bootstrapping procedure helped identify a cutoff that separated a normally distributed “low” population (∼66% of samples) from a “high” population (∼33%) of outliers. The resulting distribution of low values was normally distributed, with no skew and reasonable kurtosis. Residual plots were used to check violations of linear regression assumptions, and a Wilcoxon rank sum test was conducted when assumptions were violated. A linear mixed effects model was evaluated to assure that the correlation within a household did not significantly contribute to the data or alter the conclusions drawn from the fixed effect linear regression model. Cytometry was performed using a Beckman Coulter CytoFLEX cytometer and analyzed using FlowJo version 10 software (Tree Star). Statistical analyses were conducted using R (version 4.0.2).

### Data availability

All materials, data, and associated protocols will be available to readers upon request and without undue qualifications.

## Results

**FIGURE 1.**
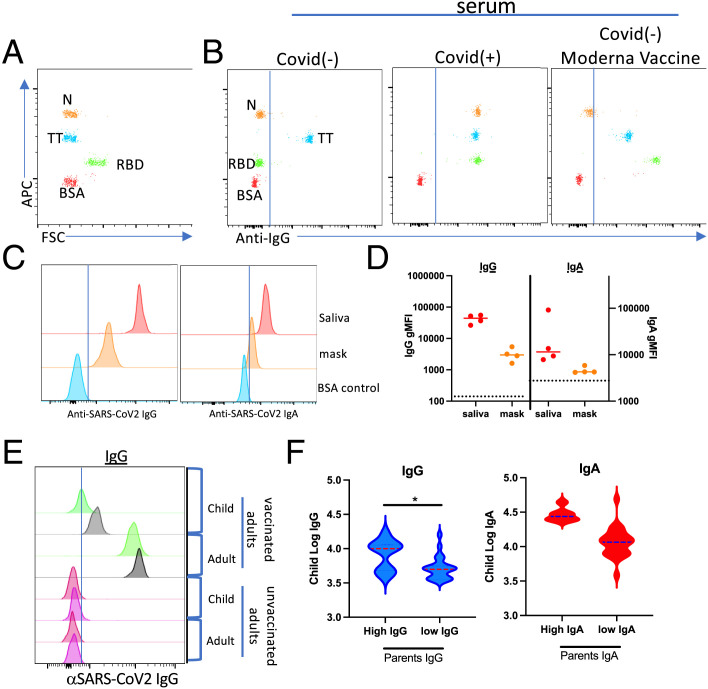
Evidence for aerosol transfer of SARS-CoV-2–specific immunity. (**A** and **B**) Representative flow cytometric results from the use of a multiplex microsphere immunoassay (MMI) (A) evaluating serum samples from a COVID-19–negative (B, left), COVID-19–positive (B, middle), and Moderna mRNA vaccinee (B, right). N, nucleocapsid protein; RBD, receptor-binding domain; TT, tetanus toxoid. Note that the TT reactivity serves as a positive control for validating sample quality in the assay. (**C** and **D**) Histograms showing the MFIs for Wuhan-RBD–specific IgG (left) and IgA (right) from saliva or eluted from a surgical mask worn for 1 work day. (D) Quantification of IgG and IgA gMFI eluted from masks obtained from four individuals. Dotted lines indicate gMFI obtained for COVID-19/vaccine^−^ sample. (**E**) Histograms showing the MFIs for Wuhan-RBD–specific IgG eluted from nasal swabs from unvaccinated children living in households in which parents or family members were either vaccinated (top) or unvaccinated (bottom). Gray and green histograms represent histograms from two separate children in whom high (gray) versus low (green) RBD Abs were identified. (**F**) Log transformation of the gMFI for Wuhan-RBD–specific IgG (left) or IgA (right) from 34 adult/child pairs using Ab cutoffs for high versus low parental intranasal Ab levels. Cutoff between adult high and low samples was determined as described in *Materials and Methods*.

The extended mandates for mask wearing in both social and work environments provided a unique opportunity to evaluate the possibility of aerosolized Ab expiration from vaccinated individuals. Utilizing a flow cytometry–based MMI to detect SARS-CoV-2–specific Abs (Fig. 1A, 1B) and a method previously used to elute Ab from rehydrated dried blood spots (3, 4), we identified anti-SARS-CoV-2–specific Abs eluted from surgical face masks that were worn for 1 work day by vaccinated laboratory members. Consistent with the results reported by others, we identified both IgG and IgA in saliva from vaccinated individuals (Fig. 1C, 1D). It was therefore not surprising to detect both IgG and IgA following elution from face masks (Fig. 1C, 1D).

Given these observations, we hypothesized that droplet/aerosolized Ab transfer might occur between individuals, much like droplet/aerosolized virus particles can be exchanged by the same route. To evaluate this hypothesis, we obtained nasal swabs from children living in households in which parents or family members had varying degrees of SARS-CoV-2–specific immunity, including those unvaccinated (anti-RBD–negative, anti-nucleocapsid protein [N]–negative), vaccinated (anti-RBD–positive, anti-N–negative), and COVID-19^+^ (anti-RBD–positive, anti-N–positive). All children evaluated were presumed to be COVID-19–negative based on being negative for anti-N Abs. Initial comparison of nasal swabs acquired from children living in vaccinated households revealed readily detectable SARS-CoV-2–specific IgG (Fig. 1E), especially when compared with the complete deficit of SARS-CoV-2–specific Ab detected in the few nasal swabs we obtained from children in nonvaccinated households. We used the variation in parents’ levels of intranasal IgG as the basis of stratification across all children’s samples. Density plots of raw and log-transformed data from 34 adult/child pairs were used to establish Ab cutoffs for high versus low parental intranasal Ab levels. Evaluation of samples in this fashion revealed that high intranasal IgG in vaccinated parents was significantly associated (*p* = 0.01) with a 0.38 increase in the log-transformed intranasal IgG gMFIs within a child from the same household (Fig. 1F). This significant positive relationship was observed using either parametric or nonparametric analysis, and adjustments for the correlation within household did not alter the conclusion. Although not statistically significant, a similar trend of elevated IgA was found in the same samples.

## Discussion

The simplest interpretation of our results is that 1) aerosol transmission of Ab can occur and that 2) the propensity for this transfer is, unsurprisingly, directly related to the amount of nasal/oral Ab found within those in the population possessing immunity. We have yet to encounter an equally parsimonious interpretation, although admittedly this does not mean one does not exist. The concept of herd immunity is a central tenant of public health vaccination campaigns. Overt blockade of infection as well as a reduction in viral transmission downstream of a breakthrough infection are widely accepted conceptual mechanisms by which vaccination-induced immunity in specific individuals protects nonimmune community members. With this in mind, it stands to reason that aerosol transmission of Abs could also contribute to host protection and represent an entirely unrecognized mechanism by which passive immune protection may be communicated.

As cross-reactivity between seasonal coronavirus spike proteins and the RBD of SARS-CoV-2 has not been observed, we do not believe that our results have been unduly influenced by subjects’ prior exposure to seasonal coronavirus. Although there is some small degree of cross-reactivity between the S2 domains of SARS-CoV-2 and the OC43 seasonal strain (5, 6), this does not extend to the S1 domain or the more limited RBD. Were this to be true, it is arguable that the course of the pandemic would have been substantially altered for the better.

Unfortunately, the difficulty in recruiting participants from unvaccinated households in conjunction with the availability of the vaccines for children under the age of 5 y rendered continued sample acquisition unsustainable. As such, we were unable to determine whether the aerosol transfer of IgA might achieve statistical significance from increased sample evaluation, nor were we able to devise any assay suitable for determining the biological relevance of the observed aerosol transfer of IgG. However, whether Ab transfer mediates host protection will be a function of exposure, and it seems reasonable to suggest, all things being equal, that any amount of Ab transfer would prove useful to the recipient host. With the documented benefits of parental vaccination in reducing the risk of infection in the unvaccinated children in the same home (7), it is tempting to speculate that aerosol-mediated Ab transfer may have possibly contributed to the reported findings. It seems likely that nasal swabs originally collected for monitoring SARS-CoV-2 transmission in this study could be repurposed for examining SARS-CoV-2–specific IgG and IgA within the vaccinated adults as well as noninfected family members, potentially providing the statistical power necessary for validating the conclusions drawn in the current study.
